# Primary low-grade extrauterine endometrial stromal sarcoma: analysis of 10 cases with a review of the literature

**DOI:** 10.1186/s12957-021-02474-1

**Published:** 2022-01-13

**Authors:** You Wu, Nan Li, Rong Zhang, Ping Bai

**Affiliations:** National Cancer Center/Cancer Hospital, Chinese Academy of Medical Sciences and Peking Union Medical College, No. 17 Pan Jia Yuan Street, Chao Yang District, Beijing, 100021 China

**Keywords:** Extra-uterine endometrial stromal sarcoma, Clinicopathological features, Complete resection, Oral hormonal therapy, Recurrence rate

## Abstract

**Background:**

This study aimed to analyze the clinical and pathological features of extrauterine endometrial stromal sarcoma (EESS) and explore an effective therapeutic regimen to reduce the recurrence rate in low-grade EESS patients.

**Methods:**

Ten LG-EESS patients who were treated at the Chinese Academy of Medical Sciences Cancer Institute and Hospital from June 1999 to June 2019 were collected and analyzed.

**Results:**

(1) Patient demographics are summarized in manuscript. Preoperative CA125 examination showed that 8 patients had a median level of 49.5 U/L (15.4–168.0 U/L). (2) All ten patients underwent tumor cytoreductive surgery. Five patients underwent optimal tumor resection and achieved an R0 resection. After the initial surgery, 7 patients who had multiple metastasis were treated with adjuvant chemotherapy, 2 patients with vaginal ESS were treated with chemotherapy and radiation therapy, and 6 patients with ER/PR positive received hormone therapy with or without chemotherapy. (2) Most EESS patients had multiple tumors. The omentum was the most commonly affected site, followed by the ovaries. (3) The median follow-up was 94 (range: 27–228) months, and recurrence was observed in 3 patients (*n* = 10, 30%) who underwent non-optimal surgery and no hormone therapy. The 5-year and 10-year DFS rates were both 70%, as shown in Fig. 2. OS was both 100% at 5 and 10 years.

**Conclusion:**

As a conclusion, EESS is a rare disease and LG-EESS has a good prognosis. Surgery remains the available treatment for patients. LG-EESS has a risk of late recurrence which requires a long-term follow-up. With a limited sample size, our study shows optimal tumor reductive surgery and adjuvant hormone therapy may significantly reduce the risk of recurrence.

## Background

Endometrial stromal sarcoma (ESS) is a malignant stromal cell tumor that originates from endometrial stromal cells, and it accounts for approximately 1% of uterine malignancies and less than 10% of uterine stromal tumors [1]. ESS usually originates in the uterus, but extra-uterine ESS (EESS), which does not involve the uterus, is also found in clinical practice. Currently, there are two theories that explain the origin of EESS. One theory is that EESS originates from the malignant transformation of endometriosis, and the other theory is that it originates from the malignant transformation of Muller cells, residual cells from embryogenesis that are widely distributed in the peritoneal and pelvic cavities [2, 3]. Malignant endometriosis lesions are mostly endometrioid adenocarcinoma or clear cell carcinoma, and EESS is extremely rare [4].

By searching PubMed for literature published between 1970 and 2016 regarding EESS, Alcazar et al. [5] found that among 76 cases of EESS that originated from endometriosis, the most commonly involved sites were ovary (44.3%), pelvic organs outside the uterus and ovaries (15.2%), sigmoid rectum (7.6%), and small intestine (7.6%). Masand et al. [6] also found that the ovaries, small intestine, peritoneum, pelvic cavity, and vagina were the most commonly affected sites. Currently, the clinical treatment for EESS is still controversial. The treatment for two classes of EESS, low-grade ESS (LG-EESS) and high-grade ESS (HG-EESS), is different, in which the former class has a preferable prognosis. Besides intervention of adjuvant therapy, tumor stage and myometrial invasion all contributed to the relapse free survival [7]. The reported overall disease-specific 5-year and 10-year survival rates are 80–90% and 70%, respectively, with a high recurrence risk [8, 9].

Here, we report the study of ten pathologic confirmed LG-EESS patients accepted adjuvant therapy with a follow-up of 27–228 months aiming to determine possible prognosis factors and suggest treatments to potentially reduce EESS relapse.

## Method

### Patients

The present study included patients with histologically proven low-grade EESS who were admitted and treated at the Chinese Academy of Medical Sciences Cancer Institute and Hospital between June 1999 and June 2019. Thirteen patients were included initially, and the clinical and pathological data were retrospectively reviewed. Ten patients were confirmed with no uterine lesions by surgery and postoperative pathology or preoperative imaging examination (pelvic MR and PET-CT); three patients with a history of uterine surgery were excluded due to difficulties to confirm uterine lesions. Patient information was obtained by searching medical records or telephone calls and questionnaires reach to primary care physicians and/or attending oncologists: age at the time of diagnosis, presenting symptoms and signs, previous history of hysterectomy (if applicable), surgical procedures, pathologic features, and recurrence and survival follow-up information. Institutional review board approval was obtained prior to the initiation of this study.

### Treatment protocol

All patients received tumor cytoreductive surgery including resection of both uterine appendages, the omentum, part of the intestinal tube, abdominal and pelvic lymph nodes, or the affected vaginal vulva according their clinicopathological characteristics. Adjuvant treatment was personalized based on the extent of the disease, medical comorbidities, and physician’s recommendation. Adjuvant treatments include chemotherapy, radiotherapy, and hormonal treatment, in combination or alone after per-treatment evaluation. The chemotherapy regimens were PEI (cisplatin + epirubicin + ifosfamide), TC (paclitaxel + carboplatin), and gemcitabine plus docetaxel. Adjuvant radiotherapy was conducted through. Hormone treatment included megestrol (Megace, progesterone derivative), letrozole (Femara, nonsteroidal aromatase inhibitor), and tamoxifen, which were used mono or combined.

### Follow-up

After the completion of the initial treatment, the follow-up was conducted with the treatment in patients. Relapse was defined as the occurrence of new measurable lesion with clinical or imaging evidence and pathologically confirmed. Disease-free survival (DFS) was defined in months as the time from the date of initial surgery to the date of disease relapse. Overall survival (OS) was calculated in months as the time from the date of initial surgery to the date of death from the disease.

### Statistical analysis

All statistical analyses were performed using SPSS 23.0 (IBM Corp, Armonk, NY). Data of the current study were presented using the mean, standard deviation, median, range, ratio, and/or frequency. The Kaplan–Meier method was used to generate survival curves and rates.

## Results

### Patient characteristics

Patient demographics is summarized in Table 1. The median age of the patients at initial diagnosis was 44.5 years (range: 33 to 66 years). Clinical manifestations mainly included lower abdominal discomfort or pain (5/10, 50%), vaginal bleeding (2/10, 20%), dysmenorrhea (1/10, 10%), and pruritus vulvae (1/10, 10%); one patient had no obvious clinical symptoms and was diagnosed during routine physical examination. Four patients had a history of uterine surgery, and pathology results indicated that they all had benign uterine lesions (uterine fibroids or adenomyosis). Preoperative CA125 examination showed that 8 patients had a median level of 49.5 U/L with range from 15.4 to 168.0 U/L, of whom 5 had elevated CA125 levels.

**Table 1:** Clinical profile of the 10 patients with LG-EESS

Parameter	Number	Percent (%)
Age at diagnosis (median, range), year	44.5	33–66
Initial clinical presentation		
Vaginal bleeding	2	20
Abd distention	3	30
Abd pain	2	20
Pruritus vulvae	1	10
Dysmenorrhea	1	10
None	1	10
History of gynecologic surgery		
TAH for leiomyoma	4	40%
CA125 (median, range), U/ml	49.5	15.4–168.0
Site of tumor		
Vaginal wall	2	20
Sigmoid	3	30
Right ovary	3	30
Vulva	1	10
Pelvis	1	10
Initial surgery		
Optimal tumor resection (R0 resection)	5	50%
LND	4	40
Stage		
I	0	0
II	2	20
III	5	50
IIII	3	30
Adjuvant treatment		
QT	2	20
HT	3	30
QT+RT	2	20
QT+HT	3	30
Follow up (median, range), month	94	27–228
Relapse	3	30
Current status		
AWD	3	30
NED	7	70

*RT* Radiotherapy, *QT* Chemotherapy, *HT* Hormone therapy, *AWD* Alive with disease, *NED* No evidence of disease

According to surgical records and pathological reports, the tumors lesion in all cases located outside the uterus, with diameters ranging from 5 cm to 20 cm, and most patients had multiple tumors (8/10, 80%) with varying degrees of adhesion to the surrounding tissues or organs. The tumors were lobulated with capsule formation. Some tumors appeared grayish white with a slightly tough texture, and some appeared grayish pink or grayish brown with a soft texture resembled fish meat. Patients with large tumors had hemorrhagic necrosis and occasional focal cystic changes. Among the eight EESS patients bearing multiple tumors, the omentum was the most commonly affected site, followed by ovaries.

**Fig. 1:**
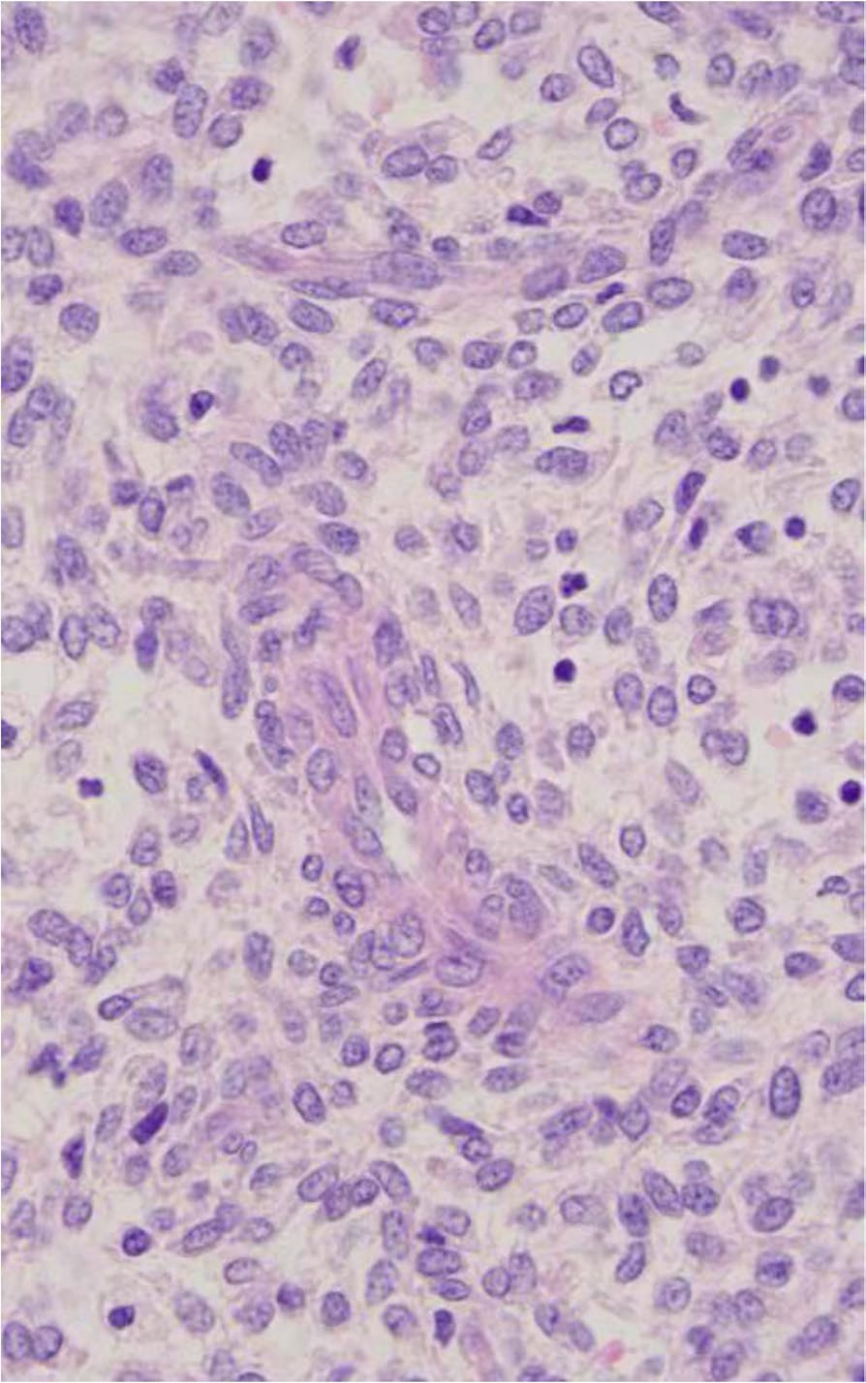
Low-grade ESS. The tumor is composed of generally uniform cells with scant cytoplasm and round to oval nuclei (original magnification × 400, hematoxylin-eosin stain)

For the morphology of low-grade EESS, most cells were small and round or in a short fusiform shape, which most were atypical, as shown in Fig. 1. The mitotic index was < 10/10HPF, and the interstitium was rich in small arterial blood vessels around tumor cells which aligned spirally. As shown in Table 2, among the 10 patients, 2 had localized smooth muscle differentiation, and 7 had definite endometriosis. In addition, 6 were estrogen receptor (ER) positive, 6 were progesterone receptor (PR) positive, and 7 were CD10 positive. Desmin was analyzed in 5 patients with 4 (80%) was negative. Smooth muscle antigen (SMA) were analyzed in 6 patients and 4 (66.7%) were negative.

**Table 2:** Pathological characteristics and immunophenotype of 10 patients with LG-EESS

Parameter	Number	Percent (%)
Tumor diameter (median, range), cm	8.7	5–20
Localized smooth muscle differentiation	2	20
Definite endometriosis	7	70
ER		
Positive	5	50
PR		
Positive	5	50
CD 10		
Positive	7	70
Desmin (*n* = 5)		
Positive	1	20
SMA (*n* = 6)		
Positive	2	33.3

*ER* Estrogen receptor, *PR* Progesterone receptor, *SMA* Smooth muscle antigen

### Surgical and adjuvant treatment

All ten patients underwent tumor cytoreductive surgery including resection of both uterine appendages, the omentum, part of the intestinal tube, abdominal and pelvic lymph nodes, and the affected vaginal vulva. Five patients underwent optimal tumor resection, with no residual tumor seen by the naked eye and achieved a R0 resection. Four patients received para-aortic LND with pelvic LND, and there was no positive observation in lymph nodes. Seven patients who had multiple metastases were treated with adjuvant chemotherapy after the initial surgery; according to the specific conditions, 2 patients with vaginal ESS were treated with chemotherapy and radiation therapy, and 6 patients with ER/PR positive received hormone therapy with or without chemotherapy. The average chemotherapy treatment duration was 6 months, 5.2 courses (range: 4–6). Three patients received only adjuvant hormonal treatment utilizing a variety of agents; the duration of medication used varied from 3 months to 1 year; by analyzing these kind of features, we can find that 2 of them underwent optimal tumor resection, the other with non-optimal surgery; meanwhile, they were all single lesion, and the DFS was 65, 77, and 66 months separately.

### Follow-up

The median follow-up was 94 (range: 27–228) months, and recurrence was observed in 3 patients (*n* = 10, 30%). By analyzing the data, 5 patients underwent non-optimal surgery, and recurrence was observed in 3 of them; meanwhile, the relapsed patients received adjuvant chemotherapy combined radiation therapy; none of them received hormone therapy. The other 2 patients had no recurrences, 1 followed by hormone therapy achieved a DFS (disease-free survival) evaluated at 66 months, and the rest followed by chemotherapy combined radiation therapy achieved a DFS at 147 months.

**Fig. 2:**
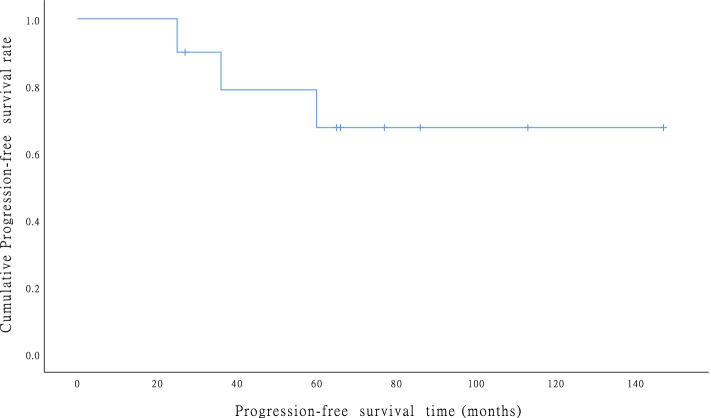
Disease-free survival (DFS). The 5-year and 10-year DFS rates for the entire cohort were both 70%

The disease-free interval of relapse was 25, 36, and 60 months, respectively. The recurrences were all developed abdominopelvic, and one patient had ureteral involvement. The 5-year and 10-year DFS rates were both 70%, as shown in Fig. 2. There was no death due to disease progression in all patients. As a result, OS was both 100% at 5 and 10 years.

## Discussion

The symptom of EESS usually involves abnormal uterine bleeding; however, an accurate diagnosis is more challenging [10]. For patients in our study, three showed abdominal distention, two with vaginal bleeding and two with abdominal pain for initial clinical appearance. To assist diagnosis, preoperative curettage and pathology, including immunohistochemistry test, are import methods [11]. LG-EESS characteristically showed positive for CD10, ER, and PR [7, 12].

In our study, small endometriosis lesions in both vaginal and vulva ESS were found which contradict with previous research [8, 9, 13]. Although there might be issues for specimen collection, a possibility that vaginal or vulva EESS involved malignant transformation of endometrial stromal cells still exists.

The treatment option for LG-EESS involved a total abdominal hysterectomy; however, ovary removal and comprehensive surgical staging including pelvic and para-aortic lymphadenectomy remained debatable [14]. In our study, the patient received radical surgery, which was uterine and double appendage resection plus tumor resection, and achieved optimal tumor reduction with a lower tumor recurrence rate.

Four patients underwent pelvic lymphadenectomy; a postoperative pathology showed negative in lymph nodes. This also supported the findings that ESS usually did not develop lymphatic metastasis [10]. We also did not directly observe the clinical benefit of lymphadenectomy which an analysis of EESS patient showed that the addition of lymphadenectomy to hysterectomy did not improve either cause-specific survival or overall survival compared to hysterectomy alone [15, 16].

For EESS patients of reproductive age, double appendage removal is also recommended [17]. However, a recent study suggested that ovary preservation did not significantly affect the overall survival of patients [15, 18]. In our study, one young patient with LG-EESS underwent fertility preservation surgery (preservation of the normal uterus and appendages) and adjuvant treatment including chemotherapy and hormone therapy. The patient did not relapse in the follow-up with a NED (no evidence of disease) which reached 86 months. Previous studies showed that two patients with vaginal EESS after local tumor resection with negative margins did not relapse in 36-month and 38-month follow-ups [8, 13]. These studies indicate that fertility preservation surgery can be performed with cautions in EESS patients who follow a desire to retain reproductive ability; however, this conclusion needs to be confirmed with large-sample-size studies.

As discussed in our manuscript, we explore the clinical features of the extrauterine endometrial stromal sarcoma; most of the cases had distant metastasis, refer to ESS; all of them were late stage, but the prognosis was excellent; OS was both 100% at 5 and 10 years. Moreover, there was no recurrence related to lymph node. The pattern of metastasis may be different from ESS; there are two theories that explain the origin of EESS. One theory is that EESS originates from the malignant transformation of endometriosis; one study found that adenomyosis is also found with an incidence of 10 to 18% in EC specimen after surgery, and EC arising from adenomyosis was associated with significantly younger onset ages and better survival than other cases where adenomyosis was just co-existing [19], which is similar with our study. And it also mentions that EC arising from, or just co-existing with, adenomyosis may be a key element to understand also the potential different responses to hormonal drugs. In our study, we also treated patients with hormone drugs, and it may reduce the recurrence rate.

Postoperative adjuvant treatment of EESS includes radiotherapy, chemotherapy, and hormone therapy [20–23]. Our study showed that postoperative radiotherapy and chemotherapy did not reduce the tumor recurrence rate, but hormone therapy, especially for patients who underwent optimal tumor reduction surgery, had significantly reduced recurrence rate compared to that of patients who did not undergo hormone therapy. As a hormone receptor positive tumor, LG-EESS was sensitive for endocrine treatment, as shown by the study [14].

Patients with LG-EESS had a good prognosis and long-term survival but with a risk of late-stage recurrence [24]. In our study, the recurrence rate was 30%, and the longest recurrence duration was 60 months.

## Conclusion

As a conclusion, EESS is a rare disease, and LG-EESS has a good prognosis. Surgery remains the available treatment for patients. LG-EESS has a risk of late recurrence which requires a long-term follow-up. With a limited sample size, our study shows optimal tumor reductive surgery and adjuvant hormone therapy may reduce the risk of recurrence, but it needs future large epidemiological studies to confirm it.

## Data Availability

All data generated or analyzed during this study are included in this published article.

## Abbreviations

ESS: Endometrial stromal sarcoma
LG-EESS: Low-grade ESS
HG-EESS: High-grade ESS
DFS: Disease-free survival
OS: Overall survival
ER: Estrogen receptor
SMA: Smooth muscle antigen
NED: No evidence of disease
LND: Lymphadenectomy
